# Solid-State Fermentation by *Trichoderma* Remodels the Metabolome and Enhances the Antioxidant Properties of Tomato Peel and Green Waste

**DOI:** 10.3390/molecules31162776

**Published:** 2026-08-10

**Authors:** Noemi Bertoli, Giorgio Gargari, Margherita Paracini, Elisa Clagnan, Emanuela Gobbi, Stefano Dall’Acqua, Gregorio Peron

**Affiliations:** 1 Department of Molecular and Translational Medicine, University of Brescia, Viale Europa 11, 25123 Brescia, Italy; noemi.bertoli@unibs.it (N.B.); margherita.paracini@unibs.it (M.P.); emanuela.gobbi@unibs.it (E.G.); 2 Department of Food, Environmental and Nutritional Sciences, University of Milan, Via Celoria 2, 20133 Milan, Italy; giorgio.gargari@unimi.it; 3 Gruppo Ricicla Lab, Department of Food, Environmental and Nutritional Sciences, University of Milan, Via Celoria 2, 20133 Milan, Italy; elisa.clagnan@unimi.it; 4 Department of Pharmaceutical and Pharmacological Sciences, University of Padua, Via Francesco Marzolo 5, 35131 Padova, Italy; stefano.dallacqua@unipd.it

**Keywords:** solid-state fermentation, *Trichoderma*, tomato peels, green waste, metabolomics, antioxidants, circular bioeconomy

## Abstract

The increasing generation of agro-industrial residues requires the development of sustainable valorization strategies for converting low-value biomasses into high-added-value products within a circular bioeconomy framework. In this study, tomato peels (TP) derived from the tomato-processing industry and green waste (GW) from urban pruning activities were investigated as substrates for solid-state fermentation (SSF) mediated by *Trichoderma harzianum*, with the aim of evaluating fungal growth, metabolomic remodeling, and the production of antioxidant bioactive compounds. Different substrate formulations containing TP and GW were subjected to SSF for 7 days, and fungal colonization was monitored. The highest fungal colonization was observed in substrates containing high proportions of GW, whereas pure tomato peels showed negligible colonization, indicating a strong substrate-dependent effect on fungal development. UPLC-QToF-MS metabolomic profiling was subsequently performed on the fermented substrate formulations using three independent biological replicates per treatment, whereas the 100% TP formulation, which showed negligible fungal colonization, was excluded from metabolomic analysis. Results revealed marked differences among fermented substrates, and 22 discriminant metabolites significantly enriched in those containing higher proportions of TP were identified. These metabolites mainly included hydroxycinnamic acid derivatives, flavonoids, lignans, phenolic glycosides, and organic acids, such as coumaric acid, hydroxycaffeic acid, cinnamoylglucose, citric acid, and cyanidin glycosides, suggesting fermentation-associated transformation and release of phenolic compounds. Fermented extracts obtained from mixed substrates enriched in TP exhibited the highest antioxidant activity, with DPPH and ABTS radical scavenging capacities reaching up to 63.48 µmol TE/g dw and 119.67 µmol TE/g dry weight, respectively, together with increased total phenolic content. The integration of microbiological, metabolomic, and antioxidant analyses demonstrated that co-fermentation of tomato-processing byproducts with green waste can modulate fermentation outcomes and enhance antioxidant potential. Overall, this study provides new insights into substrate-driven metabolic transformations during *Trichoderma*-mediated SSF and highlights the potential of mixed agro-industrial residues as sustainable feedstocks for the production of antioxidant-rich extracts with possible nutraceutical and biotechnological applications.

## 1. Introduction

The progressive intensification of agri-food production systems has generated increasing amounts of organic residues and byproducts, creating major environmental and economic challenges for their management and disposal. Among these, tomato-processing residues represent one of the most abundant waste streams generated by the food industry worldwide. According to the Food and Agriculture Organization, global tomato production exceeded 188 million tons in 2023, with China, India, Turkey, Italy, and the United States among the leading producers [1]. Italy alone processes approximately 5.8 million tons of industrial tomatoes annually, generating large quantities of pomace and secondary residues during the production of sauces, concentrates, peeled tomatoes, and canned products [2]. Tomato processing byproducts mainly consist of peels, seeds, and residual pulp, representing approximately 5–10% of the fresh fruit weight, depending on the industrial process and cultivar. Despite being commonly considered low-value waste, tomato byproducts are rich in bioactive and structurally valuable compounds, including carotenoids, phenolic acids, flavonoids, vitamins, proteins, and dietary fibers [3]. In particular, tomato peels (TP) contain significantly higher concentrations of phenolic compounds and carotenoids than the pulp, including lycopene, quercetin derivatives, rutin, chlorogenic acid, and hydroxycinnamic acids. These compounds exhibit antioxidant, antimicrobial, anti-inflammatory, and nutraceutical properties [4], making tomato residues promising feedstocks for biorefinery-oriented valorization strategies. Recent studies have highlighted their potential application in functional foods, nutraceuticals, cosmetics, animal feed, and agricultural biostimulants [5,6]. Nevertheless, the direct exploitation of tomato by-products and plant residues remains challenging because of their high moisture content, rapid perishability, compositional heterogeneity, and complex lignocellulosic matrices limiting the accessibility of bioactive compounds [7,8]. Conventional extraction approaches commonly rely on organic solvents or acid-assisted extraction systems and often require energy-intensive processing, raising concerns regarding environmental sustainability, process scalability, operator safety, and the possible presence of residual extraction chemicals that may limit the direct use of the recovered products in food, nutraceutical, cosmetic, and feed applications [9]. Consequently, increasing attention has been directed toward biological and biotechnological approaches capable of stabilizing agro-industrial residues and enhancing the recovery of high-value metabolites under milder and more sustainable processing conditions.

Among the available biotechnological strategies, solid-state fermentation (SSF) has emerged as a promising approach for the valorization of agricultural byproducts. SSF involves the growth of microorganisms on moist solid substrates under limited free-water conditions, closely mimicking the natural ecological niche of filamentous fungi. Compared with submerged fermentation, SSF offers several advantages, including reduced water and energy consumption, lower wastewater production, higher product stability, and improved enzymatic production [10,11]. Importantly, fungal SSF can promote the enzymatic degradation of plant cell walls, facilitating the release, transformation, and bioconversion of bound phytochemicals into more bioavailable or biologically active metabolites. Species of *Trichoderma* are well known for producing cellulases, hemicellulases, pectinases, esterases, and oxidative enzymes involved in plant biomass deconstruction and phenolic compound release from lignocellulosic matrices [12]. Further value for SSF is brought by the genus’ rapid colonization ability, environmental adaptability, and capacity to produce a broad range of secondary metabolites [13]. In addition to their industrial relevance, *Trichoderma* spp. are recognized producers of biologically active metabolites with antioxidant, antimicrobial, plant-growth-promoting, and biostimulant properties [14,15,16].

Recent studies demonstrated the feasibility of using fungal fermentation to enhance the recovery of antioxidant compounds from tomato-processing residues. For example, Mendez-Carmona et al. reported that fermentation of tomato byproducts with *Aspergillus niger* GH1 promoted enzymatic disruption of the plant matrix and improved carotenoid release [17]. Similarly, fungal fermentation has been shown to increase the abundance of hydroxycinnamates, flavonoids, and other phenolic derivatives through enzymatic hydrolysis and microbial biotransformation reactions [18]. Nevertheless, most available studies focused on single-substrate fermentations and targeted extraction of specific compounds, while limited information is available regarding substrate-driven metabolic remodeling during fungal SSF of mixed agro-industrial residues. In this context, lignocellulosic green residues derived from urban pruning activities represent another underexploited biomass resource. This green waste (GW) is generated in large amounts from the maintenance of public parks, gardens, and roadside vegetation. Such residues are rich in structural polysaccharides and fibrous material, potentially providing improved aeration, physical support, and carbon sources during SSF processes. In previous studies, our group demonstrated that green pruning residues and other agro-industrial byproducts can support *Trichoderma* growth and enzymatic production during SSF, with associated metabolomic remodeling and enrichment in bioactive compounds [11,19]. Co-fermentation strategies based on complementary substrates may therefore improve process stability while modulating microbial metabolism and metabolite production.

The biochemical complexity of SSF systems requires advanced analytical approaches capable of comprehensively characterizing fermentation-induced metabolic changes. In particular, as already shown in previous studies by our group [11,19], metabolomics enables the simultaneous monitoring of primary and secondary metabolites generated during SSF, providing mechanistic insights into fungal metabolism, enzymatic degradation pathways, and the formation of bioactive compounds. Despite the increasing application of metabolomics in food and microbial biotechnology, the metabolomic characterization of agro-industrial substrates transformed by SSF remains largely unexplored. To date, no study has investigated *Trichoderma*-mediated co-fermentation of TP and GW, nor how substrate composition drives metabolomic remodeling and antioxidant output in this system. This represents a significant knowledge gap, given the large volumes of tomato-processing byproducts and green waste generated annually in Mediterranean agro-industrial systems, and the recognized potential of *Trichoderma*-based SSF as a low-cost, scalable strategy for their valorization within circular biorefinery frameworks. We hypothesized that the chemical and structural composition of the fermentation matrix, determined by the relative proportions of TP and GW, would modulate the interaction between *Trichoderma harzianum* and the substrate, resulting in substrate-dependent differences in fungal growth, metabolomic profiles, and antioxidant capacity. To test this hypothesis, we investigated the effect of different TP/GW formulations on *T. harzianum* SSF by integrating fungal growth analysis, untargeted metabolomics, antioxidant assays, and multivariate statistical analyses. This integrated approach enabled us to evaluate how substrate composition influences fungal colonization, metabolomic remodeling, and the antioxidant properties of fermented substrates during SSF.

## 2. Materials and Methods

### 2.1. Byproducts and Fungal Strain

TP were obtained as a food industry byproduct from Conserve Italia Soc. Coop. Agricola (San Lazzaro di Savena, Bologna, Italy). The material consisted of industrial tomato processing residues, primarily composed of peel fractions derived from thermal peeling processes. The GW consisted of pruning residues and mixed municipal green waste, collected from Brescia municipality, Italy. All byproducts were stored at −20 °C immediately after collection and prior to use to prevent microbial degradation and metabolic alteration.

Prior to SSF processing, substrates were thawed and pretreated to ensure homogeneous moisture distribution. TP was thawed under microwave defrost conditions for 5 min, resulting in a final moisture content of 74%. GW material was hydrated by soaking 100 g of substrate in 5 L of tap water for 15–16 h at room temperature, followed by manual draining using a stainless-steel sieve to remove excess water, yielding a final moisture content of 73%. These pre-hydration steps were performed to standardize water activity across substrates and improve fungal colonization efficiency during SSF.

The fungal strain used in this study was *T. harzianum*, maintained in the microbial collection of the Agri-food and Environmental Microbiology Platform (PiMiAA), University of Brescia. For routine propagation, the strain was cultivated on slants of potato dextrose agar (PDA; Merck KGaA, Darmstadt, Germany) at 26 °C in the dark for 7 days. Plates showing active mycelial growth and sporulation were used for conidial suspension preparation. Conidia were harvested by adding 1 mL of sterile distilled water to each plate and gently scraping the surface with a sterile loop to detach the spores. The resulting suspension was filtered through sterile gauze to remove mycelial fragments and standardized to a final concentration of 10^7^ conidia/mL, as determined by haemocytometer counting under a light microscope (model B-383PH, Optika Microscopes, Ponteranica, Italy). Each fermentation unit was inoculated with 100 µL of the conidial suspension, corresponding to an inoculum of approximately 10^6^ conidia per microbox [19,20].

### 2.2. Substrate Preparation and Solid-State Fermentation

SSF experiments were designed to evaluate the effect of different lignocellulosic substrate compositions on *Trichoderma* growth and metabolite production. TP and GW were used as the main substrates and were combined in different weight-based proportions to generate a compositional gradient of fermentation matrices: 100% TP, 100% GW, 75% TP/25% GW, 50% TP/50% GW, and 25% TP/75% GW. Each substrate mixture was thoroughly homogenized by manual mixing for 5 min to ensure uniform distribution of components prior to fermentation setup. Given the structural and compositional heterogeneity of lignocellulosic matrices, particular attention was paid to achieving macroscopic homogeneity of the TP/GW blends before portioning, in order to minimize inter-unit variability in substrate composition and moisture distribution. For each experimental condition, 20 g of wet substrate were placed into micropropagation boxes (Microbox, Micropoli, Milano, Italy) equipped with a 0.45 µm gas-permeable filter to allow controlled gas exchange while minimizing contamination risk. The boxes were partially sealed with aluminum foil covering approximately three-quarters of the lid opening, while the caps were loosely positioned to further ensure passive aeration during fermentation. Each micropropagation box, already filled with the respective substrate formulation, was subjected to two consecutive autoclave cycles (121 °C, 15 min each) to ensure complete sterilization of both the container and the substrate. Boxes were then allowed to cool under a laminar flow hood for approximately 16 h before fungal inoculation.

SSF was initiated by inoculating each microbox with 100 µL of a freshly prepared conidial suspension of *Trichoderma* at a concentration of 10^7^ conidia/mL, corresponding to an initial inoculum load of approximately 10^6^ conidia per fermentation unit. Uninoculated controls were prepared for each substrate formulation and treated with an equal volume of sterile distilled water under identical conditions. For each substrate formulation, independent fermentation units were prepared for sampling during fermentation monitoring and for end-point analyses.

Fermentation was carried out in a controlled climatic chamber (Binder Model 720, Tuttlingen, Germany) at 26 °C under 60% RH with a 12 h light/12 h dark photoperiod provided by daylight fluorescent tubes (24 W/m^2^). The process was conducted under static conditions without mechanical agitation to simulate natural solid-state microbial colonization and to preserve substrate structural integrity. Following the SSF procedure developed by our group and previously described [11,19,20], fermentation was carried out for 7 days.

Fungal growth was monitored by destructive sampling at days 1, 3, 5, and 7. At each sampling time point, one independent fermentation box per substrate formulation was aseptically collected and used for CFU enumeration. From each sampled box, 1 g of fermented material was aseptically collected for microbiological analysis.

At the end of fermentation (day 7), three independent fermentation boxes per substrate formulation were harvested separately for qPCR-based fungal biomass quantification, metabolomic profiling and antioxidant analyses. Each sample was independently homogenized, aliquoted, extracted, and analyzed, providing three biological replicates (n = 3) per substrate formulation. Following homogenization, aliquots intended for DNA extraction were stored at −80 °C until analysis, whereas aliquots designated for aqueous extraction were processed as described below.

### 2.3. Fungal Biomass Quantification

Fermented material (1 g) was suspended in 100 mL of sterile deionized water and stirred for 30 min under magnetic agitation to facilitate fungal detachment from the substrate matrix. Serial tenfold dilutions were prepared and plated on PDA medium. Three replicate plates were prepared from each sample using 100 μL aliquots of the appropriate dilution. Plates were incubated at 26 °C and monitored after 24 and 48 h for colony development. Because fungal colonization in GW-containing substrates frequently exceeded the upper quantification limit of the plating method (>10^8^ CFU/g), CFU enumeration was used as a semi-quantitative indicator of fungal development and expressed using the following categorical scale: − (no detectable growth), + (moderate growth), ++ (extensive growth), and +++ (growth exceeding the quantification limit of the assay). To obtain a quantitative estimate of fungal biomass at the end of fermentation, qPCR analysis was performed on day-7 samples, corresponding to the fermentation endpoint and to the stage at which *Trichoderma* biomass is expected to reach maximal development under the experimental conditions employed, as previously reported by our group [11,19].

qPCR was performed targeting the *Trichoderma* calmodulin gene using the TCal primer set previously described by our group [20]. The fermented material was homogenized by grinding under liquid nitrogen using a mortar and pestle to obtain a fine and homogeneous powder. Total DNA was extracted from approximately 40 mg of homogenized material using the DNeasy^®^ PowerSoil^®^ Pro Kit (Qiagen, Hilden, Germany) according to the manufacturer’s instructions. DNA purity was assessed spectrophotometrically using a NanoDrop 1000 instrument (Thermo Fisher Scientific, Waltham, MA, USA) by evaluating A260/A280 and A260/A230 absorbance ratios, while DNA concentration was determined using a Qubit 1× dsDNA Broad Range Assay Kit (Thermo Fisher Scientific, Waltham, MA, USA). DNA integrity was verified by electrophoresis on 1% (*w*/*v*) agarose gels prepared in 1× TAE buffer. The sensitivity of the qPCR assay was determined by serial 2-fold dilution of genomic DNA extracted from pure *T. harzianum* cultures to define the lower limit of detection. Quantification cycle (Cq) values obtained from experimental samples were interpolated against a standard curve to estimate the amount of *Trichoderma* spp. DNA present in each sample, and appropriate conversion factors were applied to calculate the gene copy number per gram of substrate (GCC/g). qPCR data were expressed as the mean ± standard deviation (SD) of the independent DNA extractions. Statistical differences in fungal genomic abundance among substrate formulations were assessed by one-way ANOVA followed by Tukey’s post hoc test, with significance set at *p* < 0.05, using GraphPad Prism 8.0.1.

### 2.4. Aqueous Extraction of Fermented Samples and Antioxidant Assays

After 7 days of SSF, fermented and control substrates were subjected to aqueous extraction to recover extracellular and soluble intracellular metabolites associated with fungal activity and substrate transformation. For each substrate formulation, the material obtained from three independent fermentation boxes was processed separately. Each fermented sample was individually homogenized before extraction. Then, approximately 20 g of homogenized material were suspended in 100 mL of sterile deionized water (substrate-to-solvent ratio of 1:5, *w*/*v*). The mixture was stirred at 200 rpm for 20 min at room temperature to facilitate the diffusion of polar metabolites into the liquid phase. Following homogenization, the slurry was filtered under vacuum through Miracloth (Merck Millipore, Darmstadt, Germany) to remove solid particulate matter and obtain a clarified aqueous phase. The filtrate was then transferred to sterile centrifuge tubes and centrifuged at 13,000× *g* for 15 min to further eliminate fine suspended particles and microbial debris. The resulting supernatant was carefully collected and passed through a 0.45 µm membrane filter (Merck Millipore, Darmstadt, Germany) to ensure removal of residual biomass and obtain a particle-free extract. The clarified extracts were aliquoted, lyophilized using a L-250 freeze-drier (BÜCHI Labortechnik AG, Flawil, Switzerland), and stored at −20 °C until further analysis.

The antioxidant capacity of the lyophilized aqueous extracts was evaluated using two complementary radical scavenging assays, namely 2,2-diphenyl-1-picrylhydrazyl (DPPH) and 2,2′-azino-bis(3-ethylbenzothiazoline-6-sulphonic acid) (ABTS), together with the determination of total phenolic content (TPC) as an estimate of the overall phenolic load. The DPPH assay was performed by assessing the ability of the extracts to quench the stable DPPH radical through hydrogen or electron donation, and results were expressed as µmol Trolox equivalents (TE) per gram of dry extract (µmol TE/g dw). Similarly, the ABTS assay was conducted by measuring the capacity of the extracts to reduce the ABTS•+ radical cation generated through chemical oxidation, with results also expressed as µmol TE/g dw. Both radical scavenging assays were performed following the method reported in the literature [21]. Trolox (Merck, Darmstadt, Germany) was used as the reference standard for both assays, and calibration curves were constructed under linear response conditions. Statistical comparisons of extracts from fermented and control substrates were performed by two-way ANOVA in GraphPad Prism 8.0.1, comparing SSF mean with control mean for each substrate. Correction for multiple comparisons was performed using the Bonferroni test, and the confidence interval was set at 95% (significance level: *p* < 0.05). All measurements were performed in triplicate and results are expressed as mean ± SD.

TPC was determined using the Folin–Ciocalteu method, based on the reduction in the phosphomolybdic-phosphotungstic acid complexes under alkaline conditions [22]. Briefly, aliquots of appropriately diluted extracts were mixed with Folin–Ciocalteu reagent (Merck, Darmstadt, Germany) and allowed to react before the addition of sodium carbonate solution to develop a blue chromophore. The reaction mixture was incubated in the dark at room temperature for 60 min, and absorbance was measured spectrophotometrically at 765 nm. Gallic acid (Merck, Darmstadt, Germany) was used as the calibration standard (calibration curve: y = 0.012x + 0.058, R^2^ = 0.999), and results were expressed as mg gallic acid equivalents (GAE) per gram of dry extract (mg GAE/g dw). All measurements were performed in triplicate and results are expressed as mean ± SD. Statistical comparisons among treatments were performed using two-way ANOVA, followed by Bonferroni post hoc testing, with significance set at *p* < 0.05.

### 2.5. Metabolomic Profiling of Extracts from Fermented Substrates

Untargeted metabolomic profiling of aqueous extracts was performed using an ultra-performance liquid chromatography system (UPLC Acquity H-Class, Waters, Milford, MA, USA) coupled to a Xevo QToF mass spectrometer (Waters) equipped with an electrospray ionization (ESI) source operating in negative ion mode (ESI−). Chromatographic separation was achieved using a Waters BEH C18 column (2.1 × 100 mm, 1.7 µm particle size) maintained at 40 °C, with a binary mobile phase composed of 0.1% formic acid in water (A) and 0.1% formic acid in acetonitrile (B), delivered at a constant flow rate of 300 µL/min. The elution gradient was programmed as follows: 0–1 min, 98% A; 11 min, 15% A; 16–20 min, 0% A; 21–24 min, 98% A, ensuring efficient separation of polar and semi-polar metabolites. The injection volume was 2 µL. To evaluate system stability and data reproducibility, a pooled quality control (QC) sample was prepared by mixing equal aliquots from every individual sample extract. QC samples were injected periodically and randomly distributed throughout the run sequence. Mass spectrometric acquisition was performed over a mass range of 50–2000 Da, with a sampling cone voltage of 40 V, source offset of 80 V, capillary voltage set at 2.5 kV, nebulizing nitrogen gas at 800 L/h, and desolvation temperature at 400 °C. Mass accuracy and reproducibility were ensured by continuous infusion of leucine–enkephalin as lock mass via a Lockspray interface at 20 µL/min, with automatic real-time correction of *m*/*z* values during acquisition. Fragmentation data were acquired in MS^e^ mode using a fixed collision energy of 30 V to generate both precursor and product ion information for structural elucidation of metabolites.

Raw LC-MS data were processed using MarkerLynx XS software Version 4.2 (Waters), where chromatographic alignment, peak detection, and deconvolution were performed to generate a structured data matrix. Processing parameters included a retention time window of 1–20 min, mass tolerance of 0.01 Da, noise elimination level set at 5, minimum intensity threshold corresponding to 15% of base peak intensity, a maximum of 6 ions per retention time window, and a retention time tolerance of 0.01 min. Isotopic peaks were excluded from further statistical analysis, and only reproducible ion features detected across samples were retained, while variables with >80% missing values were removed from the dataset. The resulting processed matrix consisted of aligned retention time–*m*/*z* pairs, sample identifiers, and corresponding ion intensities.

### 2.6. Untargeted Metabolomics and Coerrelation Analysis

Prior to multivariate statistical analysis, LC-MS data were exported to MetaboAnalyst 6.0 (https://www.metaboanalyst.ca) (accessed on 29 June 2026), where they were sum-normalized, log-transformed, and Pareto-scaled to reduce technical variability and enhance biological signal detection. An exploratory Principal Component Analysis (PCA) was first performed to assess analytical robustness; the tight clustering of pooled QC samples at the center of the PCA score plot confirmed analytical consistency and instrument stability throughout the run. Partial Least Squares Discriminant Analysis (PLS-DA) was subsequently used to visualize global metabolic differences among treatments, while hierarchical clustering (HC) was applied to assess similarity patterns among samples and metabolite profiles. Discriminant metabolites contributing to group separation were identified using one-way analysis of variance (ANOVA), applying a false discovery rate (FDR)-corrected significance threshold of *p* < 0.05. Significant features were further evaluated by Fisher’s least significant difference (LSD) post hoc test to identify differences among individual treatments and to compare fermented samples with their corresponding uninoculated controls. The resulting metabolite set was used for HC, heatmap construction, and boxplot visualization of normalized peak areas.

Metabolite annotation was performed following the Metabolomics Standards Initiative (MSI) guidelines and assigned a putative identification level (Level 2), based on accurate mass measurement, isotopic distribution, and MS/MS fragmentation spectra obtained from MS^e^ acquisition. Molecular formula prediction was conducted using the Elemental Composition tool (Waters), and candidate compounds were selected based on a mass error tolerance of ≤10 ppm. Putative identifications were assigned by database searching against PubChem (https://pubchem.ncbi.nlm.nih.gov/), KNApSAcK (https://www.knapsackfamily.com/KNApSAcK/) (accessed on 29 June 2026), and the Food Metabolome Database (https://foodb.ca/) (accessed on 29 June 2026), complemented by literature comparison. Final annotations were supported by evaluation of fragmentation patterns derived from MS^e^ spectra.

To explore the relationship between metabolomic profiles, fungal abundance, and antioxidant properties, Spearman’s rank correlation analysis was performed using MetaboAnalyst 6.0. Prior to analysis, datasets were log-transformed and Pareto-scaled to reduce heteroscedasticity and enhance comparability across variables. Spearman’s rank correlation coefficients (ρ) and corresponding *p*-values were calculated to evaluate monotonic relationships between metabolite abundances and antioxidant parameters (DPPH, ABTS, and TPC), where values of ρ < 0 indicate inverse relationships, ρ > 0 indicate positive relationships, and values approaching |1| indicate stronger associations. Statistical significance was set at *p* < 0.05. Correlation patterns were visualized as a heatmap combined with hierarchical clustering, allowing identification of metabolite groups positively or negatively associated with fungal colonization and antioxidant performance across the different substrate formulations.

## 3. Results and Discussion

### 3.1. Effect of Substrate Composition on Trichoderma Colonization and Fermentation Feasibility

**Figure 1:**
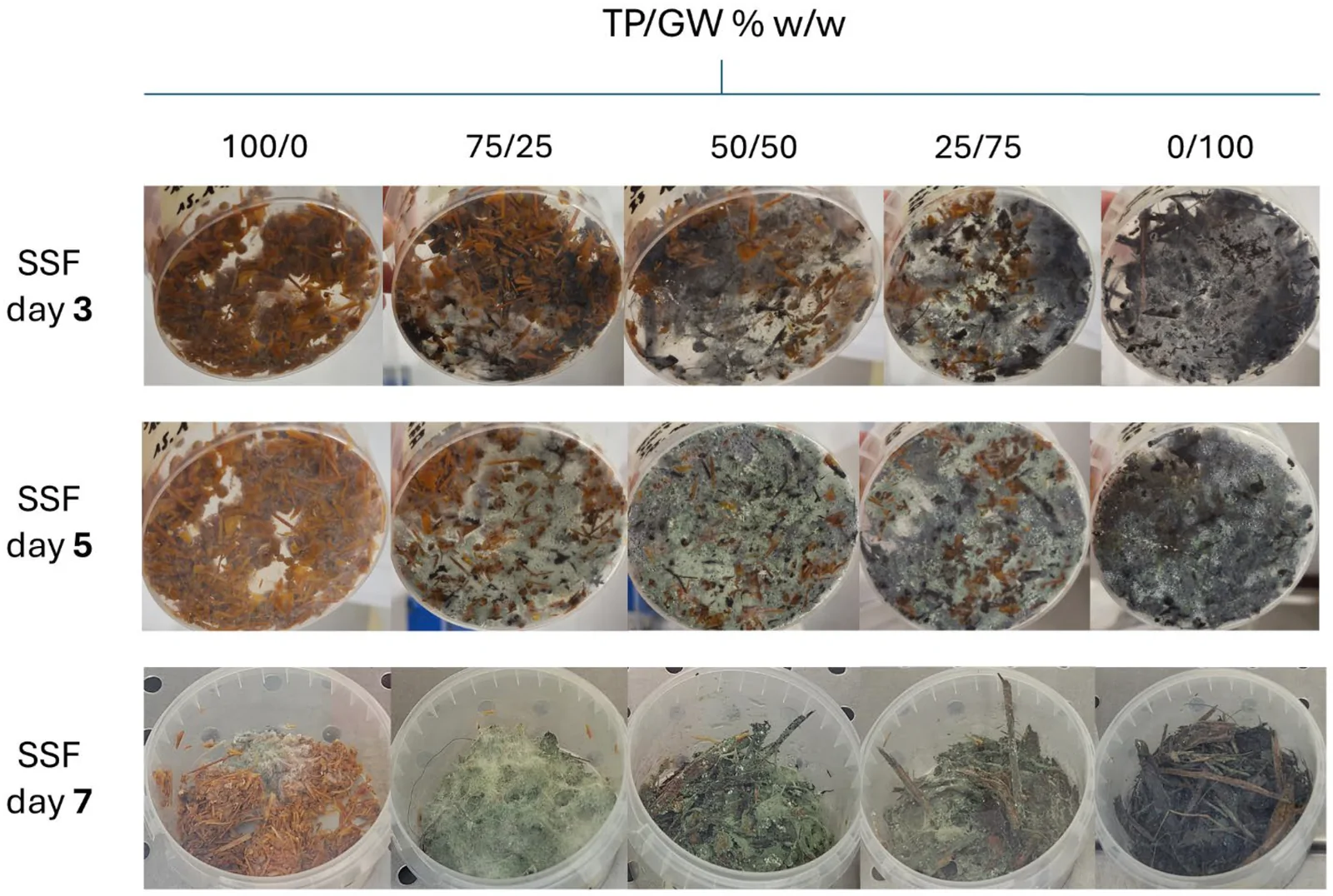
*Trichoderma* biomass after 3, 5, and 7 days of SSF on substrates at different tomato peels (TP)/green waste (GW) compositions.

A progressive increase in fungal proliferation was observed throughout fermentation, with maximal colonization detected after 7 days of SSF (Figure 1). Semi-quantitative CFU monitoring data across all substrate formulations and time points are reported in Supplementary Table S1. Substrates containing GW alone or in combination with TP showed extensive fungal proliferation at days 5 and 7, with colony development exceeding the upper quantification limit of the plating method (>10^8^ CFU/g) and precluding absolute enumeration. In contrast, no detectable fungal growth was observed in the substrate composed exclusively of TP, confirming the strong inhibitory effect exerted by this matrix on *Trichoderma* development. Interestingly, despite the reduced fungal growth observed in TP-rich substrates, mixed formulations still supported substantial viable fungal proliferation, indicating that partial substitution of GW with TP did not completely impair fungal establishment.

Quantitative molecular analysis confirmed the strong influence of substrate composition on fungal growth (Table 1). The highest *Trichoderma* colonization was detected in the 100% GW substrate, with GCC values ranging from 17,761 to 70,453 GCC/g, and was significantly higher than in all TP-containing formulations (*p* < 0.05). Although fungal abundance decreased with increasing TP proportion, pairwise comparisons among TP-containing formulations were not significant in the overall ANOVA (*p* > 0.05), likely because the high variability observed in the 100% GW group increased the residual variance. To further evaluate this trend, a one-way ANOVA was performed excluding the 100% GW group. This analysis identified a significant difference between the 25% TP/75% GW and 75% TP/25% GW formulations (*p* = 0.0465), whereas the comparisons between 25% TP/75% GW and 50% TP/50% GW (*p* = 0.0553) and between 50% TP/50% GW and 75% TP/25% GW were not significant. These results support a TP-dependent reduction in *Trichoderma* colonization, with the clearest difference observed between the lowest and highest TP-containing mixed substrates.

The substrate composed exclusively of TP exhibited inconsistent fungal detection by qPCR, with most replicates yielding DNA concentrations below the detection threshold and only one replicate showing detectable amplification (6276 GCC/g). Two explanations should be considered. First, this pattern may reflect an unstable and heterogeneous fungal colonization in the 100% TP matrix, consistent with the strong inhibitory effect of tomato-derived phytochemicals on *Trichoderma* growth demonstrated by CFU enumeration. Second, the TP matrix is known to accumulate high concentrations of polyphenolic compounds, cuticular waxes, and acidic metabolites that are well-documented co-purification contaminants during nucleic acid extraction from plant-rich matrices [3,23]. These compounds can reduce DNA yield and purity and directly inhibit polymerase activity during PCR amplification, generating false-negative or inconsistent qPCR signals independently of the actual fungal biomass present. Consequently, the absence of reproducible qPCR signal in 100% TP replicates should be interpreted cautiously: while it is consistent with limited fungal colonization, it may also partially reflect matrix-associated analytical underestimation.

The progressive decline in fungal abundance with increasing TP content suggests that TP-derived substrates imposed physicochemical or biochemical constraints on fungal development. TP are characterized by high concentrations of phenolic compounds, glycoalkaloids, flavonoids, and acidic metabolites, many of which possess antimicrobial or antifungal properties. In particular, hydroxycinnamic acids, coumaric acid derivatives, ferulic acid conjugates, and flavonoids such as quercetin derivatives have been extensively associated with antifungal activity and oxidative stress induction in filamentous fungi [24,25].

The observed inhibition of *Trichoderma* growth in TP-rich substrates is particularly noteworthy because it contrasts with the commonly reported enhancement of fungal growth and phenolic release during SSF of agro-industrial residues. Several studies have shown that *Trichoderma*-mediated fermentation increases phenolic accessibility and antioxidant activity through enzymatic degradation of lignocellulosic structures and liberation of bound phenolics. For example, *T. reesei* fermentation of garden cress seeds increased total phenolic content by approximately nine-fold and significantly enhanced antioxidant activity [26], while solid-state cultivation of *T. harzianum* on soybean matrices enhanced both phenolic abundance and antioxidant properties through modulation of polyphenolic compounds [27]. However, these studies generally involved substrates with lower intrinsic antimicrobial pressure and higher carbohydrate accessibility than TP residues. Indeed, tomato processing byproducts are known to contain elevated concentrations of bioactive phenolics with antimicrobial properties [28], and tomato peel fibers are particularly enriched in insoluble structural polysaccharides and cuticular components that may reduce substrate accessibility and water availability during SSF [28,29]. The inhibitory effect observed in the present work therefore likely resulted from a combination of limited substrate accessibility and accumulation of antifungal phytochemicals. Beyond their direct effects on fungal growth, these substrate characteristics may also influence the physiological response of *Trichoderma*. Previous studies have shown that exposure to phenolic-rich lignocellulosic substrates can modulate the expression of genes involved in oxidative stress response [30], carbohydrate-active enzymes [31], and detoxification pathways [32], enabling fungal adaptation to chemically complex environments. Although gene expression and enzyme activities were not evaluated in the present study, the reduced colonization observed in TP-rich substrates may suggest a physiological adjustment to substrate-specific chemical constraints rather than a simple reduction in fungal metabolic activity. Further studies are required to assess this hypothesis.

The high inter-replicate variability observed in TP-rich substrates reflects the intrinsic heterogeneity of SSF systems, where oxygen diffusion, moisture distribution, and localized metabolite accumulation generate microenvironmental gradients that strongly influence fungal colonization. Such variability is commonly reported in SSF of heterogeneous agro-industrial matrices [33,34] and remains a major challenge for process scale-up. From a biotechnological perspective, these findings indicate that TP, despite their recognized richness in bioactive compounds, may not represent an optimal stand-alone substrate for *Trichoderma*-based SSF. Conversely, blending TP with GW allowed reproducible fungal colonization, indicating that partial replacement of GW may represent a practical compromise between process feasibility and the biochemical potential of TP-rich substrates. The functional consequences of this partial colonization were investigated in the subsequent analyses.

Because reproducible fungal colonization was not achieved in the 100% TP substrate, subsequent analyses were specifically designed to investigate the biochemical consequences of successful or partially successful SSF and were therefore restricted to substrates supporting reproducible fungal activity.

### 3.2. Effect of Trichoderma-Mediated SSF on Antioxidant Activity and Total Phenolic Content

**Figure 2:**
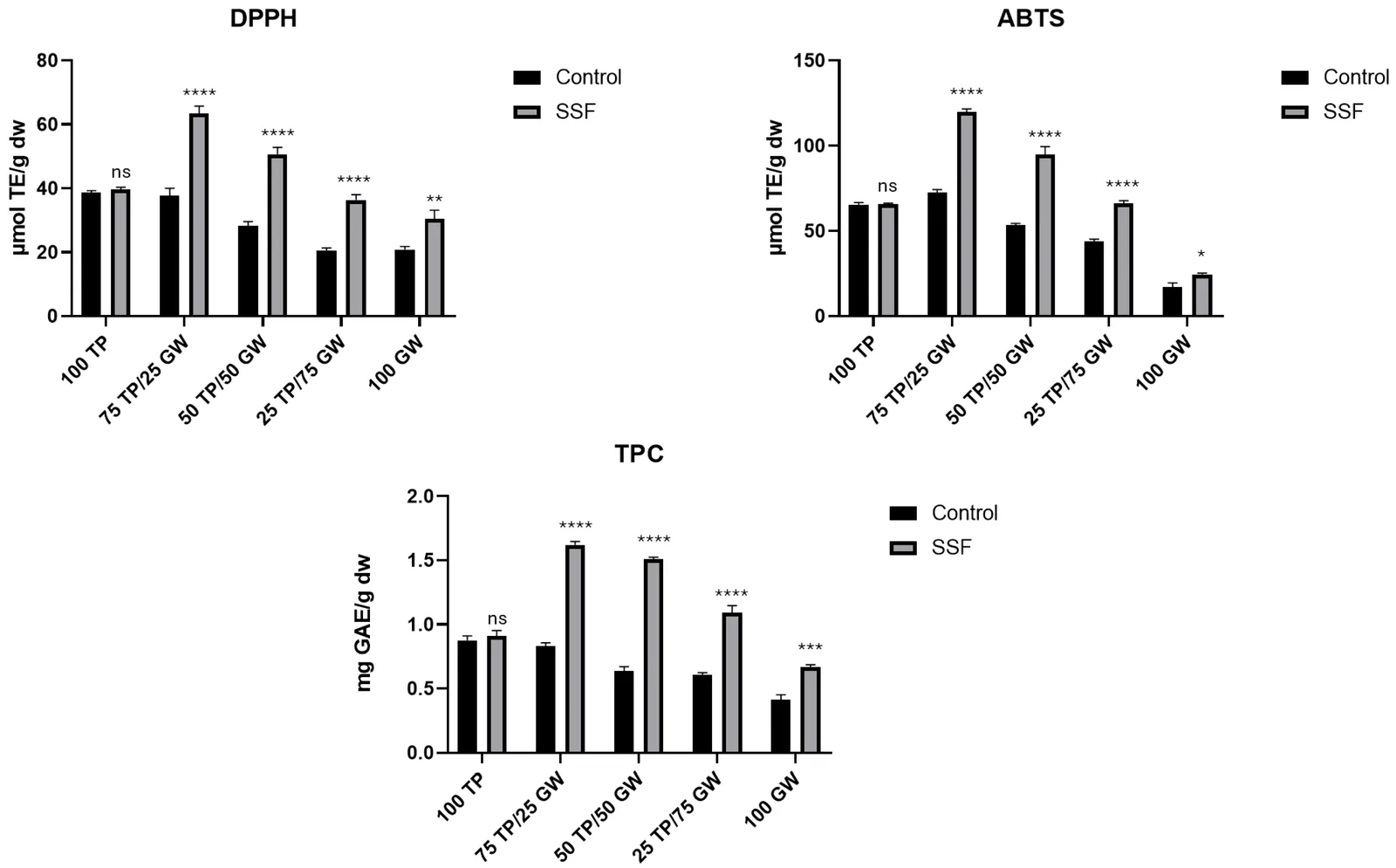
Antiradical (DPPH, ABTS) activity exerted by aqueous extracts of SSF substrates at different tomato peels (TP)/green waste (GW) compositions, and total phenolic content of the same extracts. ns: not significant; * *p* < 0.05; ** *p* < 0.01; *** *p* < 0.001; **** *p* < 0.0001.

The effects of SSF on the functional properties of substrates were evaluated by measuring radical scavenging activity (DPPH and ABTS) and TPC in aqueous extracts, thus providing information specifically on the water-soluble antioxidant fraction released or modified during fermentation. These analyses demonstrated a marked influence of substrate composition on the antioxidant properties of the fermented extracts, with TP-rich formulations exhibiting higher radical scavenging activity and TPC compared with GW-rich substrates. Comparison between fermented samples and their respective unfermented controls showed that SSF enhanced the antioxidant potential of mixed substrates, whereas only minimal effects were observed in the substrate composed exclusively of TP (Figure 2). Among all tested extracts, the one obtained from the 75% TP/25% GW substrate exhibited the highest antioxidant activity, reaching 63.48 ± 2.31 µmol TE/g dw in the DPPH assay and 119.67 ± 1.89 µmol TE/g dw in the ABTS assay, together with the highest total phenolic content (1.62 ± 0.03 mg GAE/g dw). These values were significantly higher than those observed for the extract of the corresponding unfermented control, which showed DPPH and ABTS activities of 37.73 ± 2.29 and 72.50 ± 1.82 µmol TE/g dw, respectively, and a TPC of 0.83 ± 0.03 mg GAE/g dw. Similarly, the 50:50 substrate showed a clear fermentation-associated enhancement of antioxidant properties, with DPPH activity increasing from 28.17 ± 1.49 to 50.72 ± 2.12 µmol TE/g dw, ABTS activity increasing from 53.38 ± 1.05 to 94.74 ± 4.82 µmol TE/g dw, and TPC increasing from 0.64 ± 0.04 to 1.51 ± 0.01 mg GAE/g dw following SSF. The substrate containing 25% TP/75% GW also showed an increase in antioxidant activity after fermentation, although less pronounced than in the TP-rich formulations. In contrast, the aqueous extracts obtained from the substrate composed exclusively of TP displayed only negligible differences between fermented and control samples, and similarly limited variation in ABTS and TPC values. The absence of substantial functional improvement following SSF is consistent with the microbiological analyses, indicating that limited fungal colonization constrained fermentation-dependent enhancement of the antioxidant properties of the pure TP substrate.

The relatively limited antioxidant activity detected in pure TP extracts, despite the known phytochemical richness of tomato byproducts, is partly attributable to extraction selectivity. The aqueous procedure adopted here preferentially recovered hydrophilic phenolics and glycosylated compounds, whereas hydrophobic antioxidants naturally abundant in tomato peels like lycopene, β-carotene, and non-polar flavonoid aglycones are poorly water-soluble and therefore not recovered under the extraction conditions used. Consequently, the DPPH and ABTS values reported in this study reflect exclusively the water-soluble antioxidant fraction of the fermented extracts and should not be interpreted as a measure of the total antioxidant potential of the substrate. The full antioxidant characterization of TP-rich fermented substrates, including hydrophobic fractions recoverable by organic solvent extraction, remains an objective for future investigations.

The ABTS assay consistently yielded higher values than DPPH across all samples, reflecting the broader reactivity of the ABTS radical cation toward both hydrophilic and moderately lipophilic antioxidants, as commonly reported for aqueous phenolic extracts from fermented plant matrices [35,36]. TPC generally followed the same trend as antioxidant activity, although the relationship was not strictly proportional, suggesting that qualitative differences in phenolic composition and synergistic interactions among metabolites also contributed to radical scavenging performance [37]. This observation indicates that changes in extract functionality cannot be interpreted solely on the basis of total phenolic abundance, highlighting the importance of considering compositional differences within the phenolic fraction.

Overall, SSF enhanced the antioxidant activity of aqueous extracts obtained from mixed TP/GW formulations compared with their respective unfermented controls, with the greatest enhancement observed in the 50% TP/50% GW and 75% TP/25% GW formulations despite their reduced fungal abundance. In contrast, no meaningful improvement was detected in aqueous extracts from the pure TP substrate, consistent with its inability to support reproducible fungal colonization and active metabolic activity. This behavior suggests that intermediate substrate formulations provided the most favorable balance between fermentation feasibility and phenolic availability. Whereas GW ensured sufficient fungal establishment to sustain active biotransformation, TP supplied abundant phenolic precursors that could be released or modified during SSF. Excessive TP proportions, however, appeared to limit fungal activity, probably due to the combined effects of antimicrobial phytochemicals and reduced substrate accessibility. Therefore, the higher antioxidant performance of TP-rich mixed substrates likely resulted from the combined contribution of both matrices rather than from either substrate alone. To investigate the biochemical basis underlying these functional differences, the fermented substrates were subsequently characterized by untargeted metabolomic profiling.

### 3.3. Metabolomic Profiling of Fermented Substrates and Identification of Discriminant Metabolites

**Figure 3:**
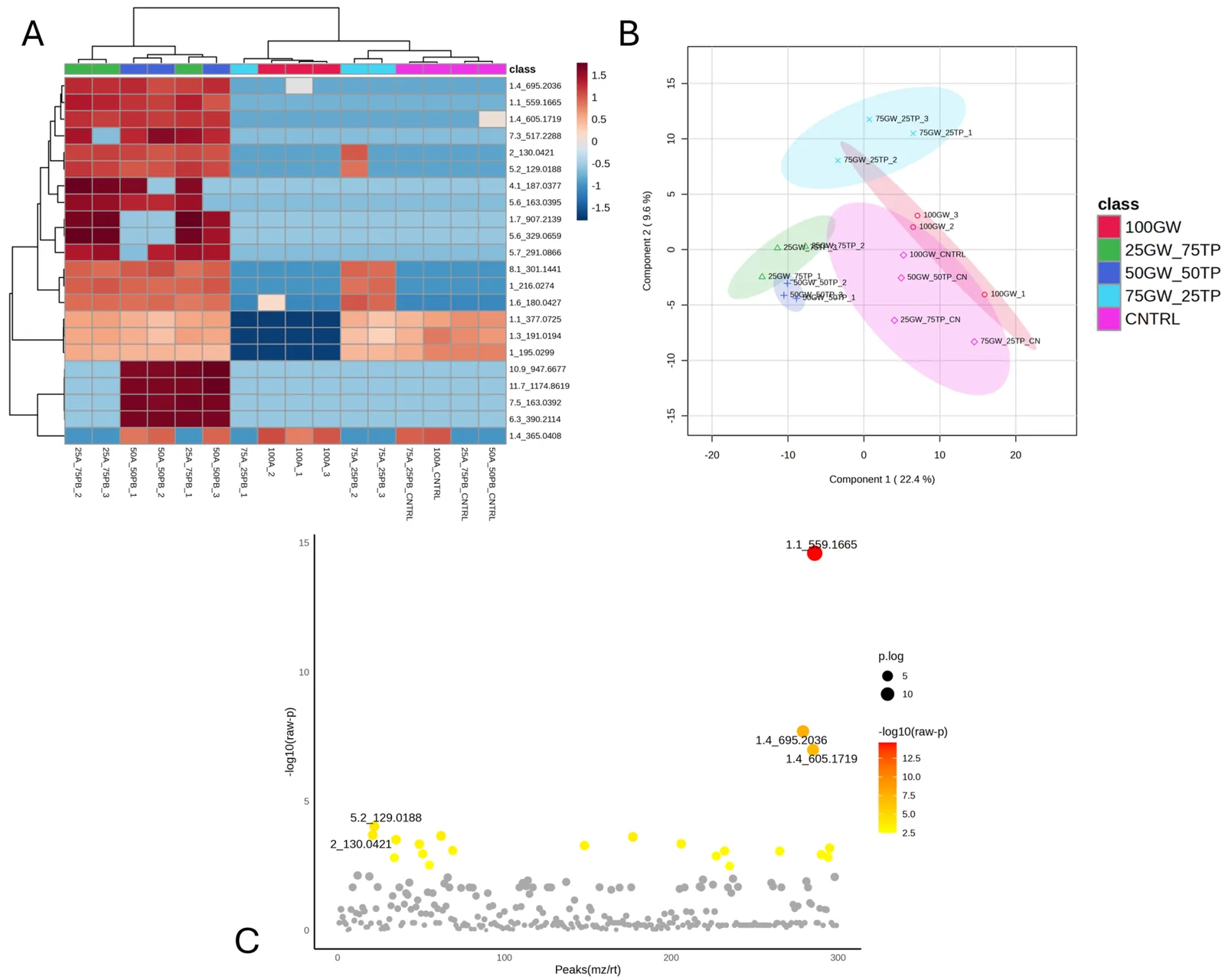
Metabolomic analysis of fermented substrates obtained after SSF with *Trichoderma*. (**A**): Hierarchical clustering heatmap based on the metabolites identified as significantly discriminant by one-way ANOVA (*p* < 0.05). Color intensity reflects normalized metabolite abundance levels; (**B**): PLS-DA score plot showing the separation of fermented and control samples according to substrate composition and metabolomic profile. Sample labels correspond to the percentage composition of green waste (GW) and tomato peel (TP); (**C**): ANOVA plot highlighting the variables significantly contributing to sample discrimination and subsequently used for hierarchical clustering and heatmap construction.

Untargeted metabolomic profiling revealed differences in the chemical composition of the fermented substrates as a function of substrate formulation, confirming that the proportion of TP strongly influenced the metabolic outcome of the SSF process. Supervised multivariate analysis performed through PLS-DA showed a clear separation among substrate groups (Figure 3), indicating substantial metabolic reprogramming associated with both substrate composition and fungal activity. In particular, uninoculated controls clustered centrally in the score plot, suggesting a relatively conserved basal metabolomic profile independent of substrate composition in the absence of fungal metabolism. Conversely, fermented substrates displayed different spatial distributions according to the relative abundance of TP and GW. Samples composed of 100% GW and 75% GW/25% TP clustered above the controls, mainly along the PC2, accounting for approximately 9% of total variance, whereas substrates enriched in tomato peels (50% and 75% TP) clustered separately on the left side of the score plot, with a markedly stronger discrimination corresponding to approximately 20% variance explained by PC1. Substrates composed exclusively of TP were not included in the metabolomic comparison, as the absence of reproducible fungal colonization prevented discrimination between substrate-intrinsic metabolites and fermentation-induced biochemical remodeling.

The observed metabolomic distribution only partially mirrored fungal abundance determined by qPCR. Indeed, although *Trichoderma* colonization progressively decreased with increasing TP content, metabolomic shifting increased in parallel, particularly in the 75% TP substrate. This observation suggests that the metabolic profile of the fermented extracts was not exclusively driven by fungal biomass accumulation, but rather by substrate-specific biochemical transformations occurring during SSF and likely involving fungal metabolic activity under stress-associated conditions. Such behavior is consistent with previous studies reporting that moderate microbial stress during SSF may enhance liberation and biotransformation of phenolic compounds through activation of oxidative and hydrolytic pathways [38,39].

To identify the metabolites contributing most significantly to sample discrimination and to assess whether their accumulation was associated with SSF rather than with the composition of the substrates, one-way ANOVA followed by Fisher’s LSD post hoc test was performed on the complete metabolomic dataset. Twenty-two metabolites were identified as significantly discriminant (*p* < 0.05; Table 2) and were subsequently used for HC and heatmap construction. Their peak areas (native and normalized) across all experimental groups are reported in Supplementary Figure S1. The post hoc analysis revealed a consistent enrichment pattern, with the majority of discriminant metabolites showing significantly higher abundances in the TP-rich fermented substrates than in both the uninoculated controls and the 100% GW substrate. In contrast, a smaller group of metabolites, including 3-dehydroshikimate, 3,3′,4,4′-tetrahydroxylignan and citric acid, displayed a more progressive response across substrate formulations, whereas only a limited number of metabolites reached maximum abundance in the intermediate 50% TP formulation. Overall, these results indicate that the metabolites responsible for sample discrimination were predominantly associated with biochemical remodeling occurring during SSF relative to the native metabolite profiles represented by the uninoculated controls, while also demonstrating that the extent of these changes strongly depended on substrate composition.

The HC heatmap revealed a coherent clustering pattern, with the majority of discriminant metabolites showing higher abundance in the substrates containing 75% TP (Figure 3).

This result indicates that TP-rich matrices promoted accumulation of a metabolome dominated by phenolic derivatives, aromatic compounds, flavonoids, lignan-related metabolites, and organic acids. Among the most discriminant compounds identified were complex hydroxycinnamate conjugates, including 5″-(4-hydroxy-(E)-cinnamoyl)-α-L-arabinofuranosyl-(1→3)-β-D-xylopyranosyl-(1→4)-D-xylopyranoside and cis-ferulic acid [arabinosyl-(1→3)-[glucosyl-(1→6)]-glucosyl] ester. These metabolites exhibited the highest discriminatory power in the ANOVA and Fisher’s post hoc analyses, supporting their main contribution to the separation of TP-rich fermented substrates from both the uninoculated controls and the GW-rich treatments. These compounds are associated with feruloylated cell-wall polymers commonly found in tomato epidermal tissues. Their enrichment is consistent with the release of bound hydroxycinnamates during SSF. Similar increases in cell wall-associated phenolic compounds have been reported in lignocellulosic substrates fermented by filamentous fungi [40,41,42], although direct evidence for the specific activity of *T. harzianum* on TP remains unavailable.

Additional phenylpropanoid-related metabolites, including coumaric acid, hydroxycaffeic acid, cinnamoylglucose, and 3-hydroxycinnamic acid, were also enriched in TP-rich substrates. Their accumulation during SSF has frequently been associated with fungal-mediated degradation of plant cell-wall structures and the release of bound phenolic compounds [40]. Moreover, *Trichoderma* species are known to actively metabolize aromatic substrates and convert plant phenolics into structurally modified derivatives with enhanced antioxidant and antimicrobial properties [43,44,45].

Flavonoid- and anthocyanin-related metabolites were also identified, including 3,5-dimethylquercetin and cyanidin 3-O-[β-D-xylopyranosyl-(1→2)-[(4-hydroxybenzoyl)-(→6)-β-D-glucopyranosyl-(1→6)]-β-D-galactopyranoside], both significantly enriched in the 75% TP substrate. Their accumulation is consistent with changes in flavonoid composition during SSF, in agreement with previous reports describing enhanced flavonoid release following fungal fermentation of agro-industrial residues [46,47,48]. From a functional perspective, these metabolites are particularly relevant because of their well-established antioxidant, anti-inflammatory, and antimicrobial activities, as well as their emerging applications as nutraceutical ingredients and agricultural biostimulants.

The metabolomic profile also included lignan-related and aromatic metabolites such as 3,3′,4,4′-tetrahydroxylignan, 3-phenylpropionate, and (R+)-3-(4-hydroxyphenyl)lactate. These compounds may derive from the transformation of aromatic amino acids and phenylpropanoid intermediates during SSF. In particular, hydroxyphenyl lactate derivatives are commonly associated with microbial catabolism of tyrosine and phenylalanine and have been reported in fermented plant matrices and microbial aromatic metabolism [49]. However, direct evidence indicating the formation of these metabolites by *Trichoderma* remains limited. Also, although fungal metabolism likely contributed to these changes, the specific biochemical pathways involved cannot be confirmed without complementary enzymatic or transcriptomic analyses. Therefore, this has to be considered as a preliminary result to be verified in future studies.

Likewise, 4-vinylphenol, another discriminant metabolite enriched in TP-rich substrates, is typically generated through microbial decarboxylation of hydroxycinnamic acids such as p-coumaric acid via phenolic acid decarboxylase activity [50]. The formation of vinylphenols is widely reported in bacterial and yeast fermentations and is considered a hallmark of active phenolic biotransformation during fermentation processes. Nevertheless, the ability of *Trichoderma* to directly produce 4-vinylphenol under SSF conditions has not yet been clearly demonstrated. Therefore, the origin of this metabolite cannot be unequivocally attributed to *Trichoderma* and requires further confirmation.

Organic acids represented another relevant class of discriminant metabolites identified in the TP-rich fermented substrates, including citric acid and mesaconic acid. The presence of citric acid is consistent with active primary metabolism during SSF and has frequently been associated with fungal bioprocesses involving lignocellulosic substrates [51]. Although organic acids are not generally considered the primary contributors to antioxidant activity compared with phenolic compounds, citric acid may indirectly influence the antioxidant behavior of complex extracts through metal-chelating activity and modulation of redox-active reactions [52]. In contrast, the biological origin and functional significance of mesaconic acid in *Trichoderma*-mediated SSF remain poorly understood.

Overall, untargeted metabolomics demonstrated that increasing TP proportions progressively reshaped the biochemical profile of the fermented substrates, inducing the accumulation of phenolic acids, hydroxycinnamates, flavonoids, lignan derivatives, and aromatic metabolites. These findings provide a mechanistic explanation for the enhanced antioxidant activity observed in the aqueous extracts of TP-rich fermented substrates, highlighting the role of metabolite composition in determining extract functionality.

### 3.4. Integrated Correlation Analysis Between Fungal Growth, Metabolome, and Antioxidant Activity

**Figure 4:**
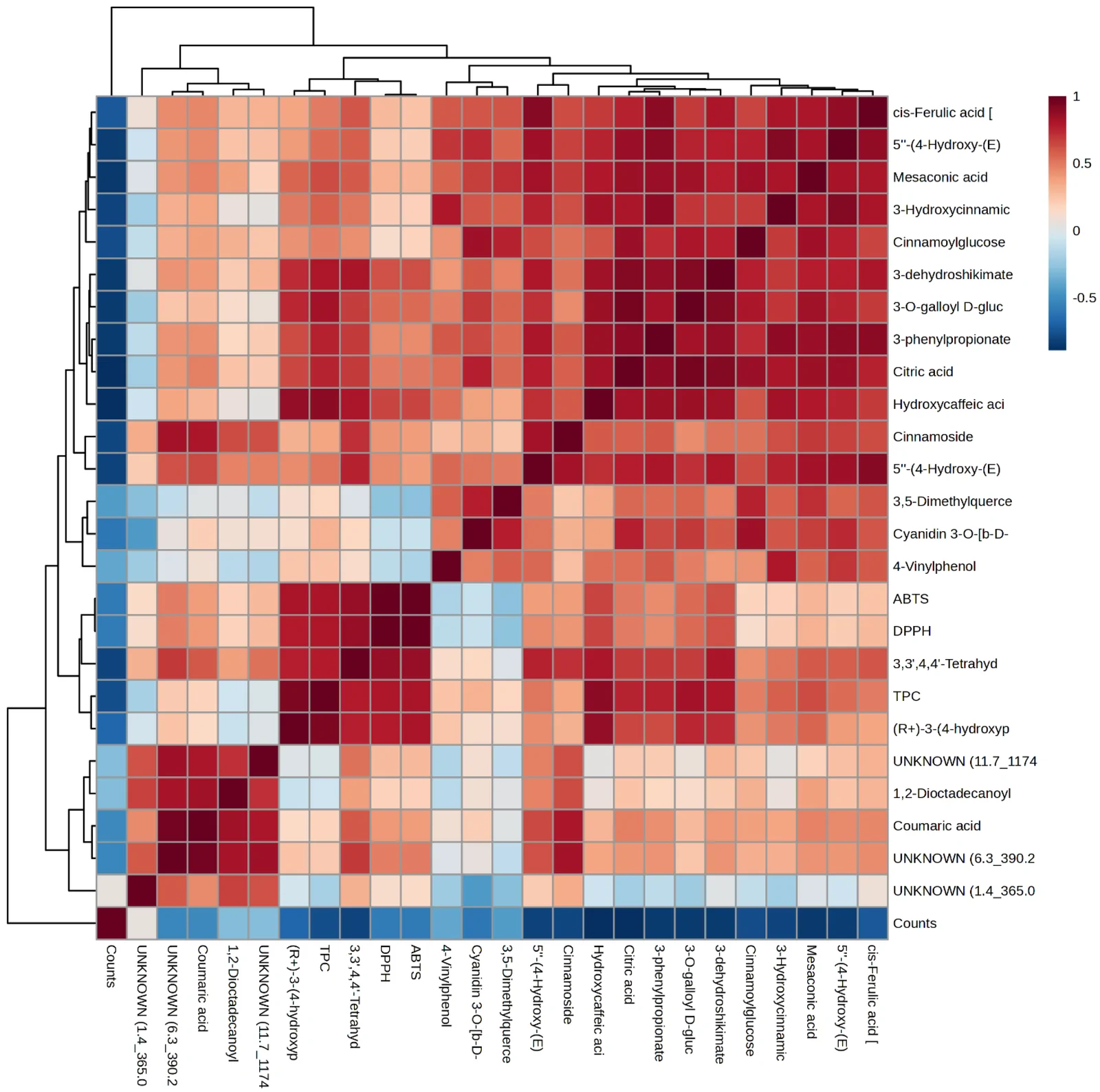
Spearman’s rank correlation heatmap between discriminant metabolites, fungal abundance, and antioxidant parameters in fermented substrates. Correlation coefficients (ρ) were calculated for all pairwise combinations of the 22 statistically significant metabolomic variables identified by one-way ANOVA (for metabolite details see Table 2), qPCR-derived fungal counts, total phenolic content (TPC), and radical scavenging activity (DPPH and ABTS). Color scale represents the strength and direction of correlations, ranging from strong positive (dark red) to strong negative (dark blue). Hierarchical clustering was applied to both rows and columns to reveal groups of co-varying variables.

The enhancement of antioxidant activity did not directly correlate with fungal abundance. Indeed, the highest *Trichoderma* colonization levels were detected in pure GW substrates, whereas maximal antioxidant activity occurred in the 75% TP formulation, which showed substantially lower fungal abundance according to qPCR analysis. Correlation analysis confirmed this inverse relationship (Figure 4).

The correlation heatmap reveals strong negative correlations between qPCR-derived fungal counts and the majority of discriminant metabolites (ρ = −0.770 to −0.887, *p* < 0.01), as well as with TPC (ρ = −0.769, *p* = 0.003) and radical scavenging activity (ρ = −0.573 with DPPH, *p* = 0.05; ρ = −0.580 with ABTS, *p* = 0.047). These results further support the observation that antioxidant enhancement was not associated with increased fungal biomass accumulation. In particular, substrates supporting the highest levels of *Trichoderma* proliferation did not correspond to those exhibiting the greatest antioxidant activity, suggesting that fungal abundance alone was not the main determinant of extract functionality. Instead, the results indicate that substrate composition and the resulting fungal-substrate interactions likely played a predominant role in shaping metabolite accumulation and antioxidant properties during SSF.

Among individual metabolites, hydroxycaffeic acid (ρ = 0.894 with TPC, *p* < 0.001), (R+)-3-(4-hydroxyphenyl)lactate (ρ = 0.932 with TPC; ρ = 0.785–0.813 with DPPH/ABTS, *p* < 0.003), and 3,3′,4,4′-tetrahydroxylignan (ρ = 0.860–0.867 with DPPH/ABTS, *p* < 0.001) showed the strongest associations with antioxidant activity. This pattern highlights that metabolites responsible for group discrimination in the PLS-DA are not necessarily the same compounds that contribute most directly to radical scavenging activity. Metabolite abundance, molecular structure, degree of conjugation, and interactions among compounds within the extract may influence their apparent contribution to antioxidant performance. Hydroxycaffeic acid may represent a plausible contributor to the antioxidant response because it belongs to the hydroxycinnamic acid family, whose free hydroxyl groups are associated with electron donation and radical stabilization. Conversely, some hydroxycinnamate conjugates identified as discriminant features may display different antioxidant behavior because glycosylation or esterification can modify their chemical accessibility and reactivity in aqueous assays [53]. Therefore, the weaker correlation observed for some discriminant metabolites does not necessarily indicate a lack of biological relevance, but may reflect differences in chemical properties, abundance, and their specific contribution to the overall extract activity. These results suggest that the enhanced antioxidant activity was associated with substrate-dependent metabolic profiles generated during SSF. Such profiles may reflect altered fungal-substrate interactions under the different chemical constraints imposed by TP- and GW-rich matrices, potentially involving differential release and transformation of polar phenolic metabolites as previously reported for fungal fermentation systems [27,54]. However, the specific enzymatic mechanisms underlying these changes and the direct contribution of individual metabolites require further validation through targeted quantification and functional assays.

Overall, *Trichoderma*-mediated SSF enhanced the functional value of mixed TP/GW substrates, while pure TP matrices were poorly suitable for efficient fungal biotransformation. These results highlight substrate formulation as a key determinant of both fungal physiology and metabolite production during SSF, and support the potential of mixed agro-industrial residues as feedstocks for the sustainable production of bioactive extracts within circular biorefinery frameworks.

## 4. Limitations and Future Directions

Several limitations of the present study should be acknowledged. First, fungal colonization in TP-containing substrates may have been partially affected by matrix-related analytical constraints. Polyphenols, cuticular compounds, and other plant-derived components present in TP can interfere with DNA extraction and PCR amplification, potentially resulting in underestimation of fungal abundance. Therefore, although qPCR indicated reduced *Trichoderma* colonization in TP-rich substrates, complementary approaches such as microscopy, biomass determination, or internal extraction controls will be useful to confirm fungal growth patterns in complex plant matrices.

Second, the interpretation of metabolomic changes is based on indirect evidence. Metabolite annotations were assigned at MSI Level 2 based on accurate mass and MS/MS fragmentation but were not confirmed using authentic standards. In addition, enzyme activity assays, transcriptomic analyses, and stable isotope tracing were not performed; therefore, direct causal relationships between specific fungal activities and metabolite modifications cannot be established. Future studies should combine targeted metabolite validation with enzyme assays and RNA-seq approaches comparing *Trichoderma* responses under TP- and GW-rich conditions to elucidate the mechanisms underlying substrate transformation.

Third, the correlation analysis integrating metabolomic variables, fungal abundance, and antioxidant parameters should be considered exploratory. Because multiple pairwise comparisons were evaluated, false-positive associations cannot be completely excluded. Therefore, the observed correlations should be interpreted as hypothesis-generating relationships requiring confirmation through targeted quantification and functional assays.

Fourth, the study was performed using a single *T. harzianum* strain and one SSF configuration. Additional strains, inoculum conditions, fermentation times, and substrate formulations should be evaluated to determine the robustness and transferability of the process. Moreover, antioxidant characterization was limited to aqueous extracts; therefore, DPPH, ABTS, and TPC measurements describe only the water-soluble bioactive fraction and do not represent the complete antioxidant potential of the biomass, including hydrophobic compounds such as lycopene and carotenoids.

Finally, scalability and economic feasibility were not assessed. Pilot-scale fermentations and techno-economic analyses will be required to evaluate process reproducibility, substrate conversion efficiency, antioxidant recovery yield per ton of residue, production costs, and comparison with existing valorization strategies. These evaluations will be essential to determine the potential application of SSF-derived extracts in fields such as nutraceuticals and agricultural biostimulants.

## 5. Conclusions

Co-fermentation of tomato-processing byproducts and green pruning residues through *Trichoderma*-mediated SSF represents a promising strategy for the sustainable valorization of agro-industrial residues within a circular bioeconomy framework. The results demonstrated that substrate composition strongly influenced both fungal development and extract functionality. GW promoted *Trichoderma* colonization and fermentation feasibility, whereas TP-rich formulations favored the accumulation of antioxidant-associated metabolites and enhanced the functional properties of the resulting extracts.

The integration of microbiological analyses and untargeted metabolomics provided insights into fermentation-associated biochemical transformations. The results demonstrated that fungal biomass accumulation and extract functionality were not directly correlated: substrates supporting the highest *Trichoderma* colonization did not correspond to those producing extracts with the greatest antioxidant activity. Conversely, mixed TP/GW formulations, particularly TP-enriched substrates, improved extract functionality despite lower fungal abundance, demonstrating that SSF performance should be evaluated considering both microbial growth and the quality of the resulting bioactive fraction.

From a biorefinery perspective, blending TP with GW represents a practical strategy to overcome the limited suitability of pure TP as a fermentation substrate while exploiting its contribution to antioxidant metabolite enrichment. These findings emphasize substrate formulation as a key parameter for balancing process feasibility and product quality in SSF systems. Although the present results are specific to the tested substrates, fungal strain, and fermentation conditions, they provide a basis for further optimization of SSF processes through targeted metabolite validation, improved extraction strategies, and scale-up evaluation.

Overall, this study demonstrates that *Trichoderma*-mediated SSF combined with metabolomics-guided characterization can transform heterogeneous agro-industrial residues into antioxidant-enriched extracts with potential applications in nutraceutical, agricultural, and biotechnological sectors.

## Figures and Tables

**Table 1:** Quantification of *Trichoderma* colonization in fermented substrates after 7 days of SSF. Values are expressed as genomic copy concentration (GCC/g substrate) reported as mean ± SD. Different letters indicate statistically significant differences among substrate formulations (*p* < 0.05). TP = tomato peels; GW = green waste.

Substrate Composition	GCC/g Substrate
100% GW	37,794 ± 28,876 ^a^
25% TP/75% GW	2505 ± 1526 ^b^
50% TP/50% GW	346 ± 124 ^b^
75% TP/25% GW	244 ± 85 ^b^
100% TP	nd †

† The substrate yielded inconsistent amplification across biological replicates, with only one replicate producing a detectable signal (6276 GCC/g); remaining replicates fell below the qPCR detection threshold.

**Table 2:** Differential metabolites identified by untargeted UPLC-QToF metabolomics analysis in fermented substrates. Metabolites are listed in the same order as the heatmap reported in Figure 3.

RT (min)	*m*/*z*	Molecular Formula	Main Fragments	Tentative Identification	Ion Type	FDR-adj *p*-Value	Compound Class
1.1	559.1665	C_24_H_32_O_15_	163.0405 131.0355	5″-(4-Hydroxy-(E)-cinnamoyl)-α-L-arabinofuranosyl-(1→3)-β-D-xylopyranosyl-(1→4)-D-xylopyranoside	[M−H]^−^	9.37 × 10^−13^	Hydroxycinnamate
1.4	695.2036	C_27_H_38_O_18_	193.0505 175.0399	cis-Ferulic acid [arabinosyl-(1→3)-[glucosyl-(1→6)]-glucosyl] ester	[M + HCOO]^−^	3.22 × 10^−6^	Feruloylated glycoside
1.4	605.1719	C_24_H_32_O_15_	163.0407	5″-(4-Hydroxy-(E)-cinnamoyl)-α-L-arabinofuranosyl-(1→3)-β-D-xylopyranosyl-(1→4)-D-xylopyranoside	[M + HCOO]^−^	6.89 × 10^−6^	Hydroxycinnamate
5.2	129.0188	C_5_H_6_O_4_	85.0299	Mesaconic acid	[M−H]^−^	0.0051	Organic acid
2.0	130.0421	C_9_H_9_O_2_	105.0717	3-Phenylpropionate	[M−H_2_O−H]^−^	0.0082	Phenylpropanoid
1.0	216.0274	C_7_H_7_O_5_	171.0282 127.0412	3-Dehydroshikimate	[M + HCOO]^−^	0.0082	Shikimate
8.1	301.1441	C_18_H_22_O_4_	ND	3,3′,4,4′-Tetrahydroxylignan	[M−H]^−^	0.0082	Lignan
5.6	163.0395	C_9_H_8_O_3_	145.0285	3-Hydroxycinnamic acid	[M−H]^−^	0.0092	Hydroxycinnamate
1.3	191.0194	C_6_H_8_O_7_	147.0293 103.0397	Citric acid	[M−H]^−^	0.0105	Organic acid
5.6	329.0659	C_17_H_14_O_7_	313.0344	3,5-Dimethylquercetin	[M−H]^−^	0.0105	Flavonoid
5.7	291.0866	C_15_H_18_O_7_	147.0450	Cinnamoylglucose	[M−H_2_O−H]^−^	0.0117	Phenolic glycoside
1.7	907.2139	C_39_H_43_O_22_	862.2159	Cyanidin 3-O-[β-D-xylopyranosyl-(1→2)-[(4-hydroxybenzoyl)-(→6)-β-D-glucopyranosyl-(1→6)]-β-D-galactopyranoside]	[M + HCOO]^−^	0.0132	Flavonoid
1.0	195.0299	C_9_H_8_O_5_	151.0405	Hydroxycaffeic acid	[M−H]^−^	0.0146	Hydroxycinnamate
1.1	377.0725	C_13_H_16_O_10_	331.0666 169.0133	3-O-galloyl-D-glucose	[M + HCOO]^−^	0.0146	Hydrolysable tannin
7.3	517.2288	C_24_H_38_O_12_	223.1332	Cinnamoside	[M−H]^−^	0.0150	Phenolic glycoside
4.1	187.0377	C_8_H_8_O	ND	4-Vinylphenol	[M−H + HCOONa]^−^	0.0171	Phenolic derivative
11.7	1174.8619	Unknown	Unknown	Unknown	Unknown	0.0183	Unidentified
6.3	390.2114	Unknown	Unknown	Unknown	Unknown	0.0197	Unidentified
10.9	947.6677	C_51_H_96_O_15_	ND	1,2-Dioctadecanoyl-3-(galactosyl-β-1→6-galactosyl-β-1)-glycerol	[M−H]^−^	0.0206	Glycolipid
7.5	163.0392	C_9_H_8_O_3_	145.0285 119.0499	Coumaric acid	[M−H]^−^	0.0208	Hydroxycinnamate
1.6	180.0427	C_9_H_9_O_4_	163.0400	(R+)-3-(4-Hydroxyphenyl)lactate	[M−H]^−^	0.0359	Aromatic organic acid
1.4	365.0408	Unknown	Unknown	Unknown	Unknown	0.0406	Unidentified

## Data Availability

Data are available upon request to the corresponding author.

## Supplementary Materials

The following supporting information can be downloaded at https://www.mdpi.com/article/10.3390/molecules31162776/s1, Figure S1: Boxplots of peak area (raw from LC-MS data and normalized) for the 22 metabolites identified as statistically discriminant by one-way ANOVA across fermented and uninoculated control substrate groups. Panels correspond to individual metabolites labeled by retention time and mass-to-charge ratio (RT_*m*/*z*; see Table 2 for compound identification details). Experimental conditions include SSF with *Trichoderma* across varying substrate mixtures: 100% GW, 75% GW / 25% TP, 50% GW / 50% TP, and 25% GW/75% TP, alongside uninoculated controls (CNTRL). TP: Tomato peels; GW: Green waste; Table S1: Semi-quantitative assessment of Trichoderma harzianum colonization across substrate formulations at days 1, 3, 5, and 7 of SSF. Fungal growth was evaluated by plating serial dilutions of substrate suspensions on PDA medium and scoring colony development after 24–48 h incubation at 26 °C. Scores are reported for three technical replicates (R1, R2, R3) and the corresponding uninoculated control (CNTRL) per substrate formulation and time point. Scoring scale: −, no detectable colony development; +, moderate fungal growth; ++, extensive fungal growth; +++, maximal fungal growth exceeding the upper quantification limit of the plating method (>10^8^ CFU/g). TP = tomato peels; GW = green waste.
