# Supplementary material for: Solid-State Fermentation by Trichoderma Remodels the Metabolome and Enhances the Antioxidant Properties of Tomato Peel and Green Waste
Source: Molecules. 2026 Aug 10;31(16):2776. doi: 10.3390/molecules31162776 (PMC13515545; doi:10.3390/molecules31162776)
Supplement: Supplementary file 1 [file molecules-31-02776-s001.zip › molecules-4440612-supplementary.pdf]

Supplementary Figure S1. Boxplots of peak area (raw from LC-MS data and normalized) for the 22 metabolites identified as statistically discriminant by one-way ANOVA across fermented and uninoculated control substrate groups. Panels correspond to individual metabolites labeled by retention time and mass-to-charge ratio (RT_m/z; see Table 2 for compound identification details). Experimental conditions include SSF with *Trichoderma* across varying substrate mixtures: 100% GW, 75% GW / 25% TP, 50% GW / 50% TP, and 25% GW / 75% TP, alongside uninoculated controls (CNTRL). TP: Tomato peels; GW: Green waste.

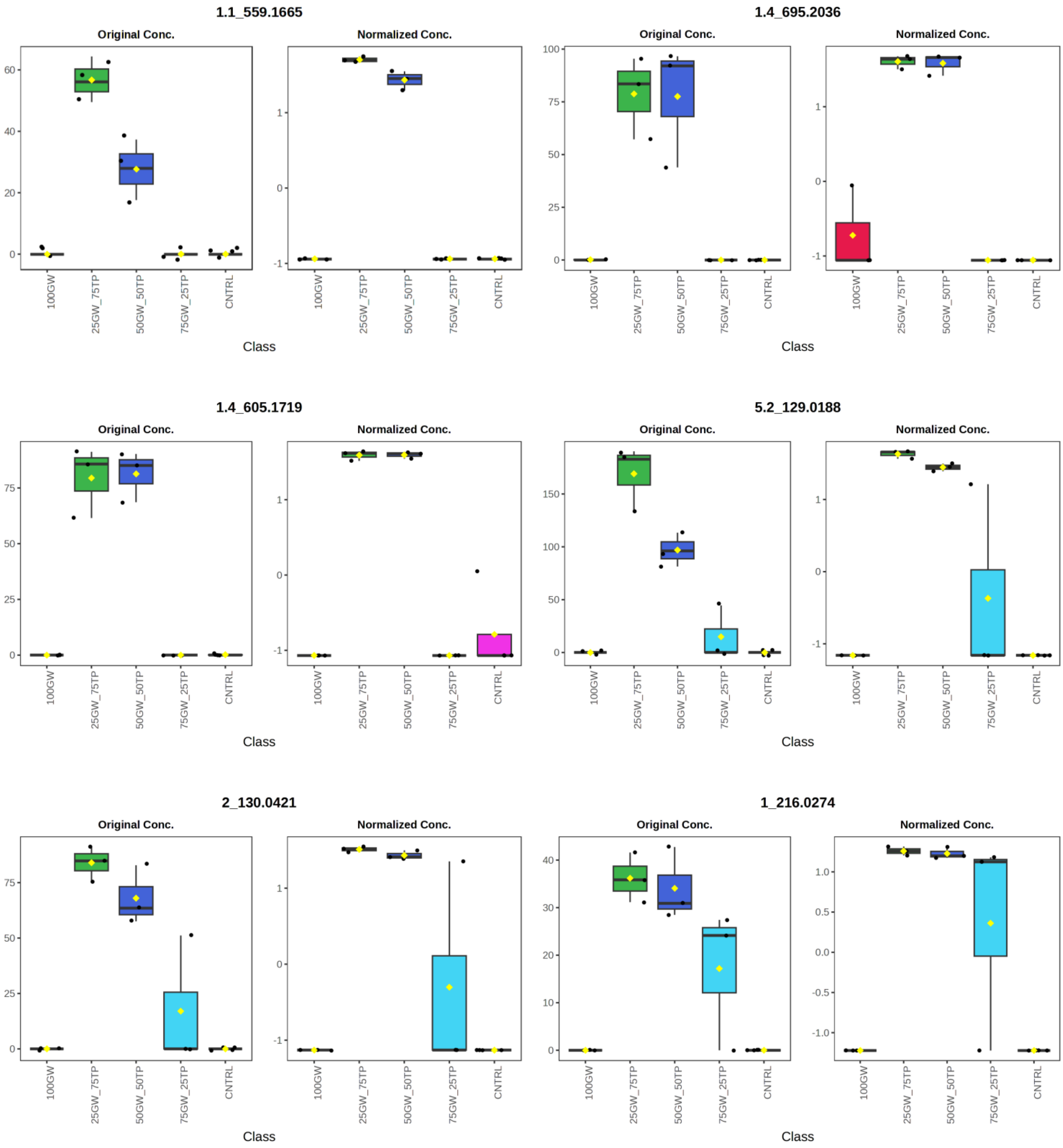

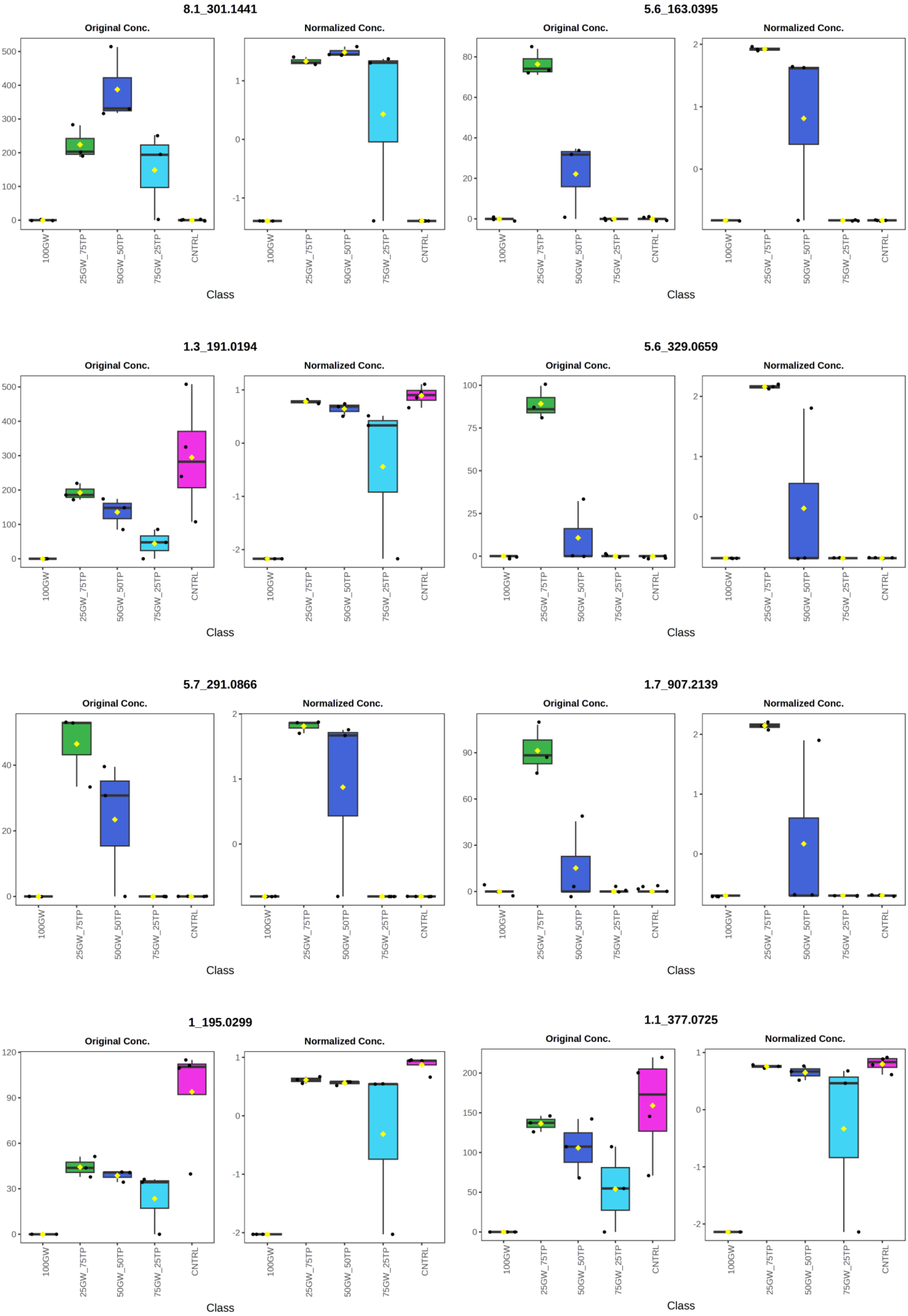
8.1_301.1441
Original Conc.
Normalized Conc.
5.6_163.0395
Original Conc.
Normalized Conc.
1.3_191.0194
Original Conc.
Normalized Conc.
5.6_329.0659
Original Conc.
Normalized Conc.
5.7_291.0866
Original Conc.
Normalized Conc.
1.7_907.2139
Original Conc.
Normalized Conc.
1_195.0299
Original Conc.
Normalized Conc.
1.1_377.0725
Original Conc.
Normalized Conc.
100GW
25GW_75TP
50GW_50TP
75GW_25TP
CNTRL
Class

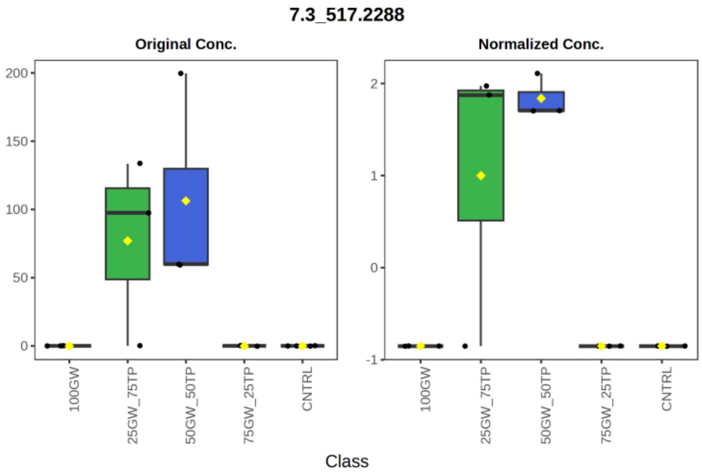
7.3_517.2288
Original Conc.
Normalized Conc.
100GW
25GW_75TP
50GW_50TP
75GW_25TP
CNTRL
Class

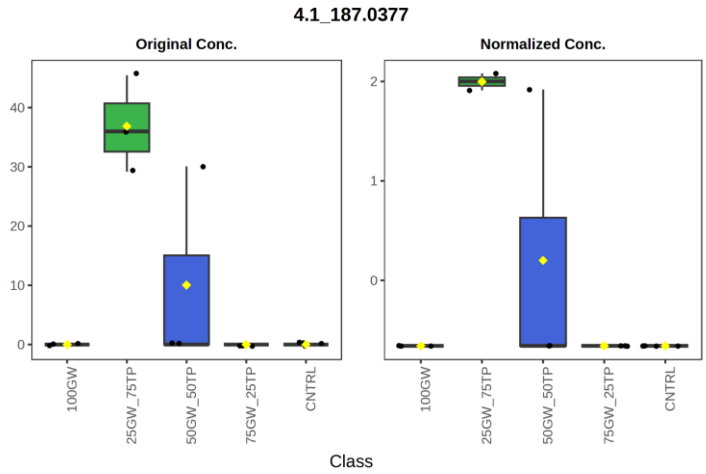
4.1_187.0377
Original Conc.
Normalized Conc.
100GW
25GW_75TP
50GW_50TP
75GW_25TP
CNTRL
Class

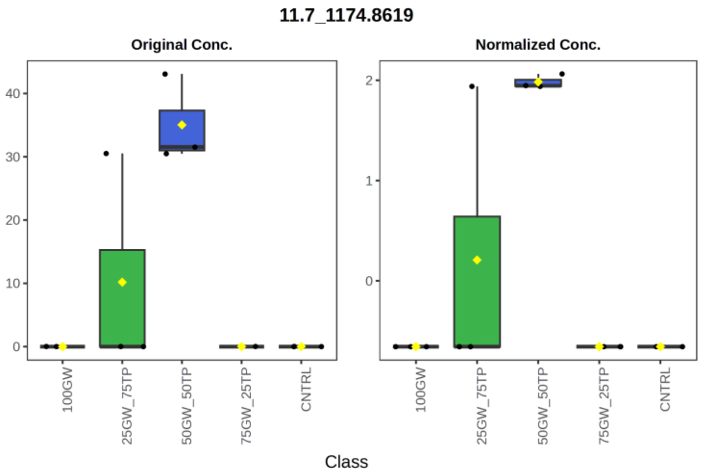
11.7_1174.8619
Original Conc.
Normalized Conc.
100GW
25GW_75TP
50GW_50TP
75GW_25TP
CNTRL
Class

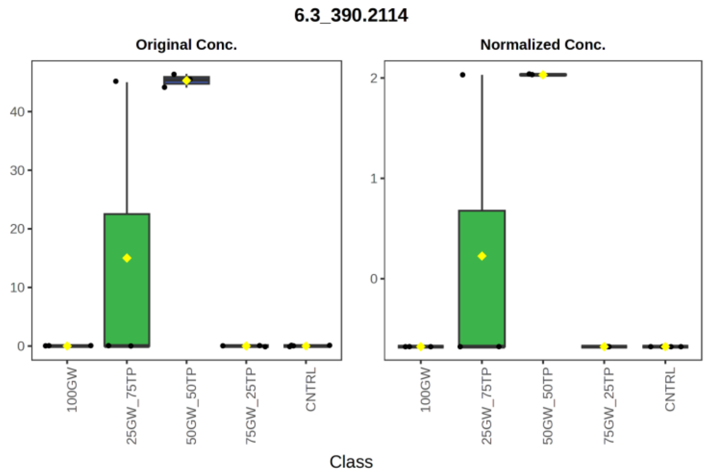
6.3_390.2114
Original Conc.
Normalized Conc.
100GW
25GW_75TP
50GW_50TP
75GW_25TP
CNTRL
Class

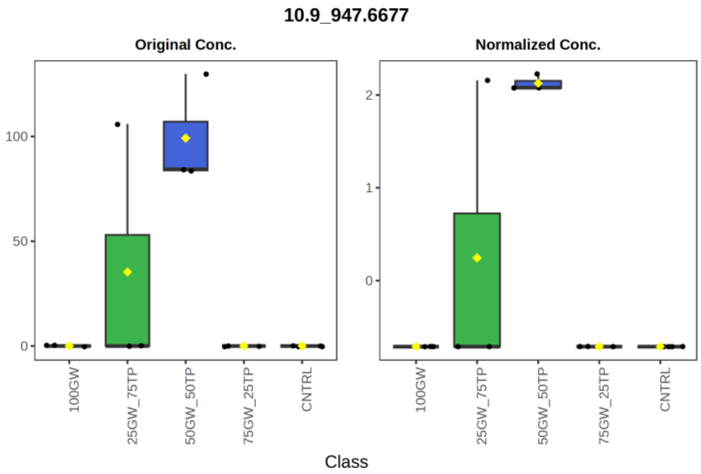
10.9_947.6677
Original Conc.
Normalized Conc.
100GW
25GW_75TP
50GW_50TP
75GW_25TP
CNTRL
Class

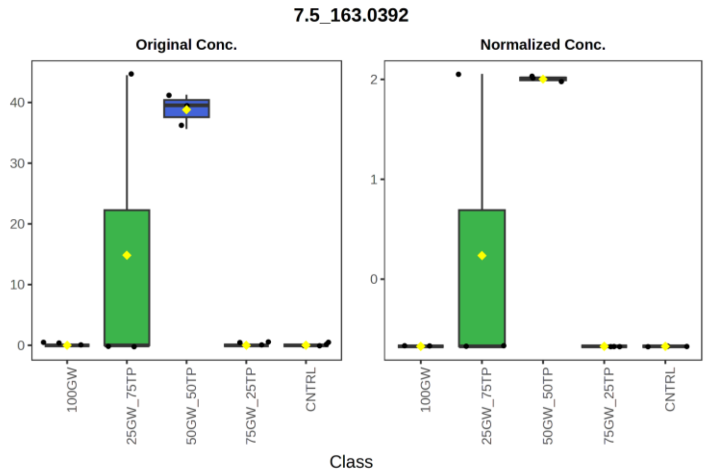
7.5_163.0392
Original Conc.
Normalized Conc.
100GW
25GW_75TP
50GW_50TP
75GW_25TP
CNTRL
Class

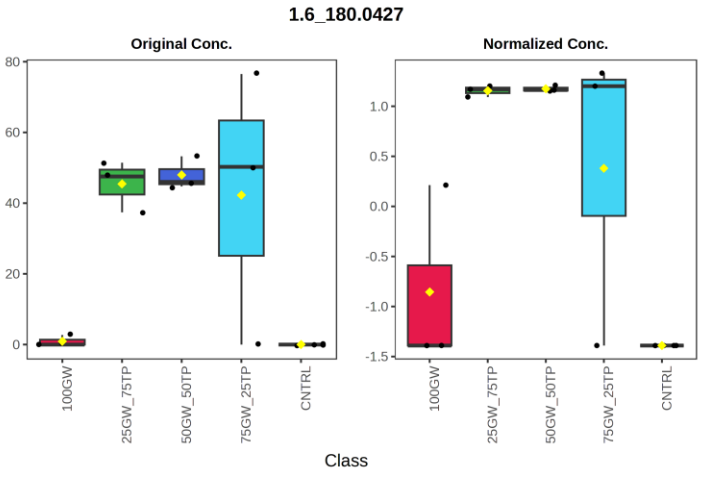
1.6_180.0427
Original Conc.
Normalized Conc.
100GW
25GW_75TP
50GW_50TP
75GW_25TP
CNTRL
Class

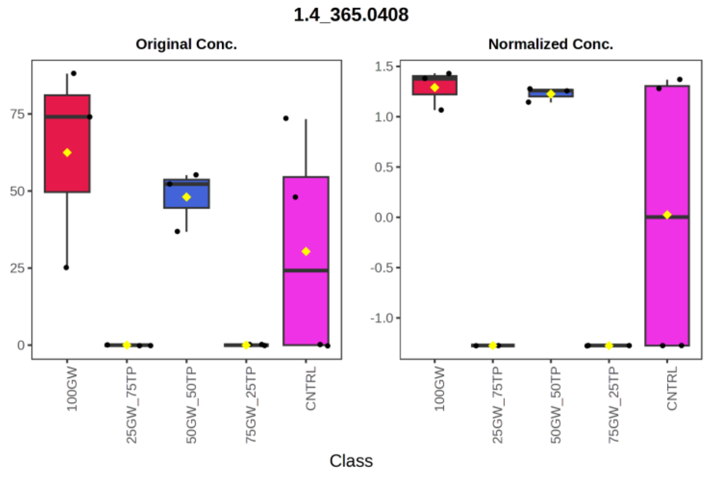
1.4_365.0408
Original Conc.
Normalized Conc.
100GW
25GW_75TP
50GW_50TP
75GW_25TP
CNTRL
Class

Supplementary Table S1. Semi-quantitative assessment of *Trichoderma harzianum* colonization across substrate formulations at days 1, 3, 5, and 7 of SSF. Fungal growth was evaluated by plating serial dilutions of substrate suspensions on PDA medium and scoring colony development after 24–48 h incubation at 26 °C. Scores are reported for three technical replicates (R1, R2, R3) and the corresponding uninoculated control (CNTRL) per substrate formulation and time point. Scoring scale: −, no detectable colony development; +, moderate fungal growth; ++, extensive fungal growth; +++, maximal fungal growth exceeding the upper quantification limit of the plating method (>10^8 CFU/g). TP = tomato peels; GW = green waste.

	**100% GW**				**100% TP**				**50% GW + 50% TP**				**25% GW + 75% TP**				**75% GW + 25% TP**			
SSF day	R1	R2	R3	CNTRL	R1	R2	R3	CNTRL	R1	R2	R3	CNTRL	R1	R2	R3	CNTRL	R1	R2	R3	CNTRL
1	-	-	-	-	-	-	-	-	-	-	-	-	-	-	-	-	-	-	-	-
3	++	++	++	-	-	-	-	-	++	++	++	-	+	+	+	-	++	++	++	-
5	++	++	++	-	-	-	-	-	+++	+++	+++	-	++	++	++	-	+++	+++	+++	-
7	+++	+++	+++	-	-	+	-	-	+++	+++	+++	-	+++	+++	+++	-	+++	+++	+++	-
